# Supplementary material for: Methyl Parathion Exposure Induces Development Toxicity and Cardiotoxicity in Zebrafish Embryos
Source: Toxics. 2023 Jan 15;11(1):84. doi: 10.3390/toxics11010084 (PMC9866970; doi:10.3390/toxics11010084)
Supplement: Supplementary file 1 [file toxics-11-00084-s001.zip › toxics-2113549-supplementary.pdf]

toxics

Supplementary Materials: Methyl Parathion Exposure Induces Development Toxicity and Cardiotoxicity in Zebrafish Embryos

Table S1. Morphological abnormalities of embryos treated with 0, 2.5 and 10-mg/L concentrations of methyl parathion at 72 hpf. A, B, C represents three repetitions per concentration.

Morphological abnormalities	Repeating group	Treatment (72hpf)			
		0 mg/L (*N*=30)	2.5 mg/L (*N*=30)	5 mg/L (*N*=30)	10 mg/L (*N*=30)
Pericardial edema	A	0 (0%)	4 (13.3%)	14 (46.7%)	26 (86.7)
	B	0 (0%)	3 (10%)	8 (26.7%)	25 (83.3%)
	C	0 (0%)	3 (10%)	10 (33.3%)	25 (83.3%)
Cardiac bleeding	A	0 (0%)	0 (0%)	2 (6.7%)	7 (23.3%)
	B	0 (0%)	0 (0%)	2 (6.7%)	9 (30%)
	C	0 (0%)	0 (0%)	0 (0%)	8 (26.7%)
Spinal curvature	A	0 (0%)	1 (3.3%)	0 (0%)	23 (76.7%)
	B	0 (0%)	0 (0%)	5 (16.7%)	25 (83.3%)
	C	0 (0%)	0 (0%)	0 (0%)	20 (66.7%)

Table S2. Sequences of primer pairs used in the real-time quantitative PCR reaction.

**Target gene**	**GenBank Acc number**	**Primer sequence (5′-3′)**	**Amplicon length**
β-actin	NM_131031.2	F: AGGTCATCACCATCGGCAAT<br>R: GATGTCCACGTCGCACTTCA	131
vmhc	NM_001112733.1	F: TGATATCGAGCGCAAACTAGCCG<br>R: CTGGATCTCCATCTCATTCAGGTCG	175
sox9b	NM_001044780.1	F: TGACGAGTTGTTCTCCAGAG<br>R: AGGCCACACGTCTATAACCC	261
nppa	NM_198800.3	F: GGATTTGACACACAGAGAAATCGCC<br>R: TTGCAGCTAACCTTTTTTAGAGTTGC	144
bmp2b	NM_131360.2	F: GAAAGCTTCGACGTGGGTTCAG<br>R: GTTCGAATGCAAGACGGCTGTG	246
bmp4	NM_131342.2	F: AGCACGTCAGCTTCGACTATCC<br>R: GGCGTCATCTATTTGTTGCCTG	209
tnnt2	NM_152893.1	F: CAGAATTGAGAAGCGTCGTTCG<br>R: TTTGCATGTAACCGCCGAAGTG	191
p53	NM_001328587.1	F: TGCTTGAAGAACAGCCTCAGCCAT<br>R: TATTCAGGTCCGGTGAATAAGTGCA	153
bax	NM_131562.2	F: GCGATACGGGCAGTGGCAATG<br>R: GCGTTTATGGCTGGGGTCACAC	158
bcl2	NM_001030253.2	F: TGGCGTCCCAGGTAGATAATATTGC<br>R: CGCAGAGGCTGTCACTTCTGAGC	235
